# Unmasking Histoplasmosis: A Diagnostic Challenge of Pediatric Febrile Neutropenia in an Immunocompromised Patient

**DOI:** 10.7759/cureus.88208

**Published:** 2025-07-17

**Authors:** Daniela Gutiérrez-Valverde, Oscar Hidalgo-Mora, Karol Acevedo-Viales

**Affiliations:** 1 Faculty of Medicine, Universidad de Ciencias Médicas (UCIMED), San José, CRI; 2 Pediatrics, Hospital Nacional de Niños, San José, CRI; 3 Hematology, Hospital Nacional de Niños, San José, CRI

**Keywords:** costa rica, disseminated, histoplasmosis, immunosuppression, pediatrics

## Abstract

Histoplasmosis is an infection caused by a dimorphic fungus. Disseminated disease in children is described mainly in infants under two years of age, but can be particularly aggressive in immunocompromised patients. We report the case of a six-year-old girl with B-cell acute lymphoblastic leukemia (ALL) who presented with fever of unknown origin, respiratory symptoms, hepatosplenomegaly, and pancytopenia. She was admitted to the hospital with febrile neutropenia and exhibited a progressively worsening clinical course. Initial work-up, including laboratory and imaging studies, failed to identify a clear infectious source. Due to signs of respiratory compromise, a chest computed tomography (CT) was performed, revealing interstitial pneumonia. A bone marrow aspirate subsequently identified intracellular budding yeast, which was confirmed as *Histoplasma capsulatum* by polymerase chain reaction* *(PCR) testing. The diagnosis of disseminated histoplasmosis was established, and treatment with intravenous liposomal amphotericin B was initiated, resulting in a favorable clinical response. The patient has now been in remission from leukemia and off treatment for one year since this infectious episode.

## Introduction

In 1905, histoplasmosis was first described as a protozoan disease in a patient from the Panama Canal [1]. Nowadays, it is known as the dimorphic fungus *Histoplasma capsulatum*, endemic to certain areas of America, Africa, and Asia. Its main reservoir is soil with chicken, pigeon, or bat droppings [2]. Its global incidence ranges between 0.1 and one case per 100,000 inhabitants per year in temperate climates, 10 to 100 cases per 100,000 in the humid tropics, and more than 100 cases per 100,000 in high-risk groups and during outbreaks [3]. The most common variety in the American continent, as well as in Costa Rica, is *H. capsulatum var. capsulatum *[4].

Infection occurs by inhalation of spores, which can disseminate through lymphatic or hematogenous routes. The infection sites may develop caseous necrosis, fibrosis, or calcification [5]. Hematogenous spread is frequently asymptomatic and self-limited. However, clinical manifestations may include fever, cough, general malaise, lymphadenopathy, visceromegaly, anemia, leukopenia, or thrombocytopenia [6]. A rare complication is secondary hemophagocytic lymphohistiocytosis (HLH), which has been described in association with disseminated histoplasmosis [7]. The degree of dissemination depends on the number of inhaled conidia and the host’s immune response [2].

In children, the clinical presentation varies with age: disseminated disease is primarily seen in infants under two years old, acute pulmonary histoplasmosis is more common in preschool-aged children, and subacute forms tend to occur in older children and adolescents. In immunocompetent children, the disease often resolves without treatment; however, in those with any degree of immunodeficiency, the course may be more aggressive [3].

Definitive diagnosis requires isolation of *H. capsulatum* in body fluids or tissue specimens. Reported detection rates achieved in blood cultures or bone marrow aspirate cultures range between 50% and 75%. Other methods of detection include antigen detection in blood or urine, serologic tests, and molecular biology techniques [8].

Recommended treatment for disseminated histoplasmosis begins with intravenous liposomal amphotericin B at 3 mg/kg/day for one to two weeks or until clinical improvement, followed by oral itraconazole at 5-10 mg/kg/day, divided into two divided doses to complete the full therapeutic course. In patients with severe disease or ongoing immunosuppression, prolonged itraconazole maintenance is advised to prevent relapse and ensure eradication of infection [9].

The pediatric population and immunocompromised patients have an increased risk of progression of the infection to its disseminated form, with a wide spectrum of clinical presentations and potential for severe complications [9]. These characteristics make early diagnosis challenging. A high index of clinical suspicion is essential to initiate timely antifungal therapy and improve outcomes.

Hence, we are reporting this case to emphasize the importance of early recognition and treatment of histoplasmosis in immunocompromised pediatric patients at high risk of complications.

## Case presentation

A six-year-old girl with B-cell acute lymphoblastic leukemia, undergoing maintenance chemotherapy and residing in a rural region of Costa Rica, was admitted to the Hematology Service with a 48-hour history of headache and diffuse abdominal pain, associated with a documented fever of 39°C, which prompted medical evaluation. Her last cycle of chemotherapy had been administered 21 days prior to symptom onset.

Initial laboratory results revealed pancytopenia and elevated inflammatory markers. Laboratory values are summarized in Table 1. The patient had a hemoglobin level of 7 g/dL, a platelet count of 17 x 10^3^/µL, and a total white blood cell count of 0.33 x 10^3^/µL, including an absolute neutrophil count of 0.19 x 10^3^/µL. C-reactive protein (CRP) was 27.4 mg/L, and procalcitonin (PCT) exceeded 100 ng/mL. Preliminary blood cultures were negative at 48 hours and remained negative after a total incubation period of five days, in accordance with institutional protocol. A nasopharyngeal swab tested positive for rhinovirus and enterovirus. Based on these findings, a diagnosis of febrile neutropenia likely due to a viral respiratory infection was suspected. Empiric broad-spectrum antibiotic therapy with ceftazidime and amikacin was initiated per institutional protocol.

**Table 1:** Key laboratory and microbiological findings during hospitalization PCT: procalcitonin; CRP: C-reactive protein; PCR: polymerase chain reaction; CMV: cytomegalovirus; EBV: Epstein-Barr virus; IGRA: interferon gamma release assay; ANC: absolute neutrophil count Reference ranges were obtained from institutional pediatric laboratory standards.

Category	Test	Patient Value	Reference Range	Units	Timing
Hematologic parameters	Hemoglobin	7	11.5–15.5	g/dL	Day 0
	Platelets	17 ×10³	150–450 ×10³	/µL	Day 0
	Total WBC	0.33 ×10³	4.5–13.5 ×10³	/µL	Day 0
	ANC	0.19 ×10³	1.5–8 ×10³	/µL	Day 0
Inflammatory markers	CRP	27.4	0–20	mg/L	Day 0
	PCT	>100	<0.5	ng/mL	Day 0
Infectious workup	Nasopharyngeal swab PCR	Rhinovirus and Enterovirus	Negative	—	Day 1
	Urine culture	MDR *E. coli* (AmpC-β-lactamase)	No growth	—	Day 5
	CMV viral load	Negative	Negative	—	Day 7
	EBV viral load	Negative	Negative	—	Day 7
	IGRA	Negative	Negative	—	Day 7
	Bone marrow aspirate (initial)	No blasts	No blasts	—	Day 10
	Galactomannan (serum)	Negative	Negative	—	Day 10
	β-D-glucan	>500	<60	pg/mL	Day 11
	*Histoplasma* PCR (urine)	Positive	Negative	—	Day 14
	*Histoplasma* PCR (bone marrow)	Positive	Negative	—	Day 14

Despite appropriate treatment therapy, the patient remained febrile and pancytopenic, requiring repeated transfusional support. Linezolid was added due to persistent fever. A urine culture subsequently isolated a multidrug-resistant *Escherichia coli* producing AmpC β-lactamases, prompting a switch to meropenem.

One week into hospitalization, the patient developed progressive respiratory distress necessitating high-flow nasal cannula oxygen therapy. Given her immunocompromised status and evolving respiratory symptoms, pulmonary aspergillosis was suspected, and empiric voriconazole was started. Bronchoscopy was deferred due to her clinical instability.

Chest and paranasal sinus CT imaging revealed bronchopneumonia without evidence of aspergillomas. Sphenoidal sinus findings were consistent with acute sinusitis, similar to a previous admission. *Pneumocystis jirovecii *pneumonia (PJP) was also considered, and a course of sulfamethoxazole-trimethoprim was initiated. A serum galactomannan assay subsequently returned negative.

Despite multiple adjustments to antibiotic therapy, the patient's clinical status failed to improve. Additional infectious workup, including cytomegalovirus (CMV) and Epstein-Barr virus (EBV) loads and an interferon gamma release assay test (IGRA) for tuberculosis, was negative. Inflammatory markers fluctuated without correlation to her condition. A bone marrow aspirate, performed to assess for leukemic relapse, confirmed the patient remained in remission.

**Figure 1:**
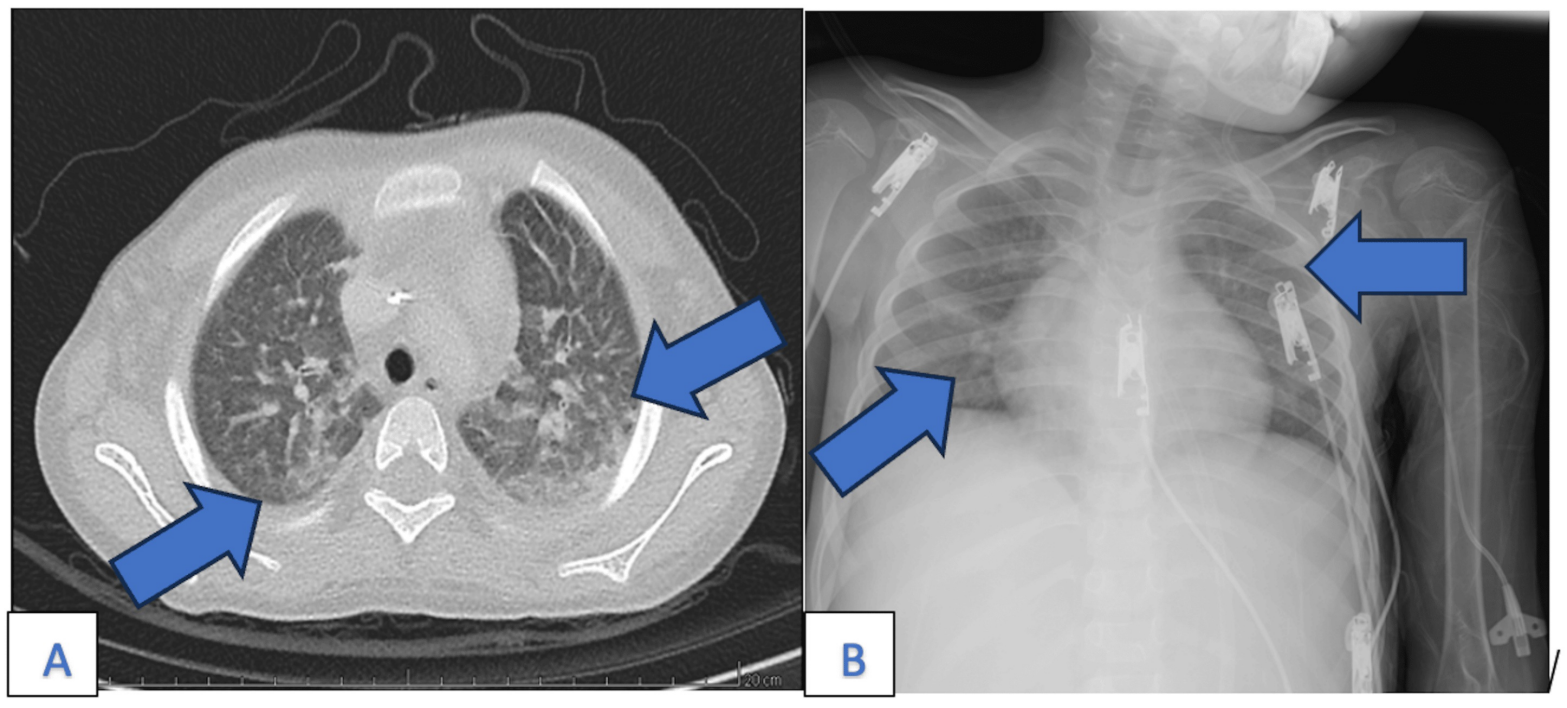
Chest CT and X-ray showing interstitial pulmonary involvement A: chest CT scan (axial view) showing bilateral diffuse ground-glass opacities (blue arrows), suggestive of interstitial pneumonia and raising concern for an opportunistic infection; B: frontal chest X-ray showing diffuse bilateral interstitial infiltrates with no focal consolidation (blue arrows). Portable imaging was limited but contributed to assessing disease monitoring.

Her clinical course continued to worsen, with new-onset hepatosplenomegaly and persistent thrombocytopenia. A serum ß-D-glucan was markedly elevated (>500 pg/mL; reference range: <60 pg/mL), raising concern for invasive fungal infection. A repeated chest CT scan showed a ground-glass pattern in the right upper lobe and pneumatoceles in the same region, suggestive of interstitial pneumonia (Figure 1A). A chest radiograph performed during clinical decompensation revealed diffuse bilateral interstitial infiltrates without focal consolidations (Figure 1B).

**Figure 2:**
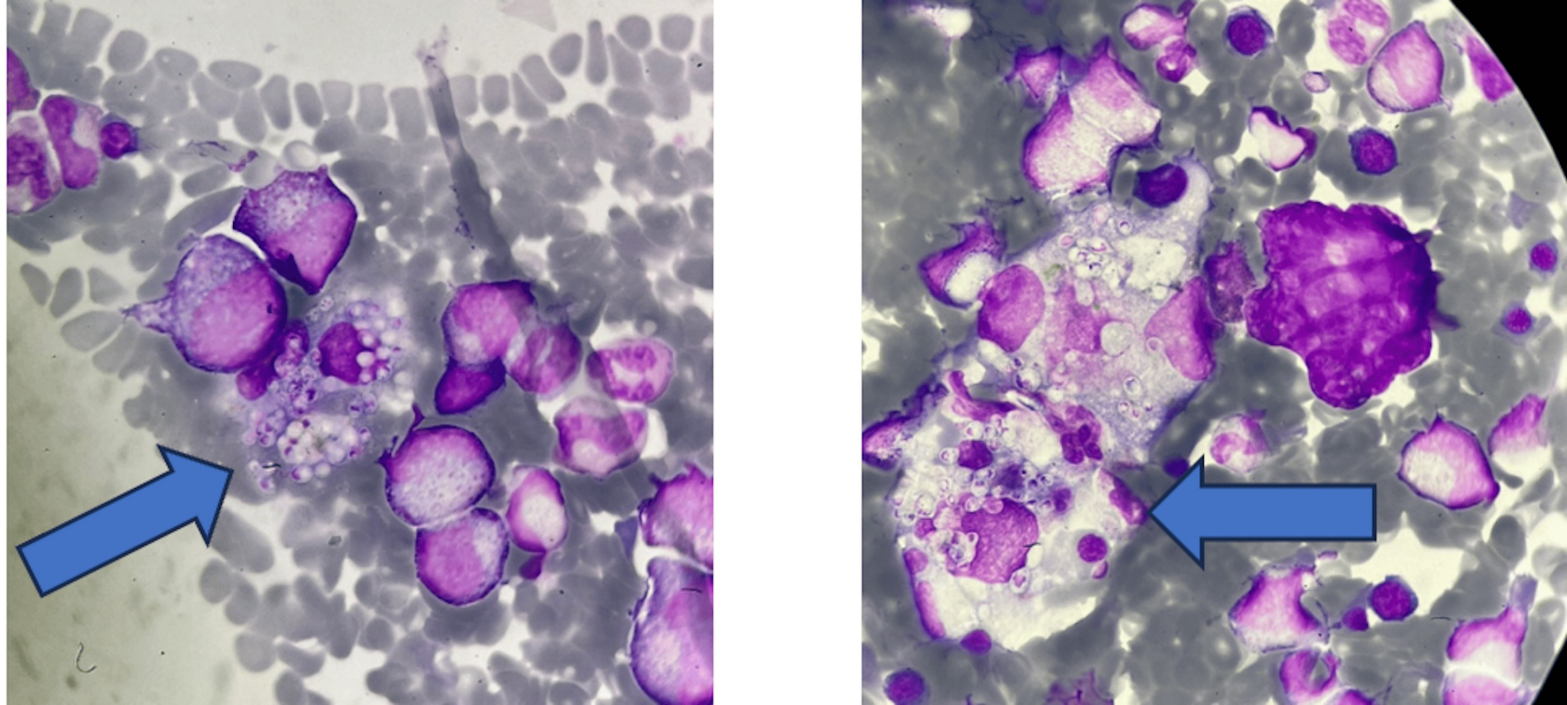
Bone marrow smear The arrows show the intracytoplasmic inclusions compatible with *H. capsulatum*. Wright-Giemsa stain, 100× magnification (oil immersion).

Prompted by these findings, the previously obtained bone marrow smear was re-examined on day 13 of hospitalization and revealed numerous cells with intracytoplasmic yeast-like inclusions, morphologically consistent with *Histoplasma capsulatum* (Figure 2). Due to her unstable condition, bronchoscopy remained unfeasible. A second bone marrow aspirate was obtained, and polymerase chain reaction (PCR) testing confirmed *H. capsulatum*. Additionally, a *Histoplasma* antigen test performed on a urine sample returned positive, further supporting the diagnosis of disseminated histoplasmosis. Of note, the patient resided in a rural region of Costa Rica, an area recognized as endemic for *Histoplasma capsulatum*. However, no specific environmental exposures, such as cave exploration, contact with bird or bat droppings, or nearby construction, were identified in the clinical history.

Intravenous liposomal amphotericin-B was promptly initiated, resulting in rapid and significant clinical improvement. After completing two weeks of intravenous therapy and once transitioned to oral itraconazole, the patient was discharged in stable condition, with a planned duration of at least 12 months. Throughout the diagnostic and therapeutic process, the Infectious Diseases team provided essential guidance and support, contributing significantly to the successful outcome of the case. 

The progression of clinical findings, diagnostic steps, and treatment decisions is summarized in Table 2. 

**Table 2:** Clinical timeline of events MDR: multidrug resistant; CRP: C-reactive protein; PCT: procalcitonin; PCR: polymerase chain reaction

Day	Event
Day 0	Admission with fever, pancytopenia, CRP↑, and PCT↑. Empiric antibiotics started (ceftazidime + amikacin).
Day 1-2	Blood cultures were negative. Respiratory viral panel positive for rhinovirus and enterovirus.
Day 5	Persistent fever. MDR* E. coli* in urine → meropenem initiated.
Day 10-12	Worsening respiratory distress → high-flow oxygen. Empiric voriconazole started. Bone marrow aspirate: no relapse. Hepatosplenomegaly + thrombocytopenia. ß-D-glucan >500 pg/mL.
Day 13	Bone marrow smear re-examined: yeast-like inclusions. PCR + urine antigen for *Histoplasma* ordered.
Day 14	Urine antigen positive. Bone marrow PCR confirmed *Histoplasma*. IV liposomal amphotericin B initiated.
Day 28	Completed 14 days of amphotericin B. Transitioned to oral itraconazole. Discharged stable.

## Discussion

Histoplasmosis is a systemic fungal infection caused by *Histoplasma capsulatum*, a dimorphic fungus endemic to several geographic regions, including parts of Central America. Transmission occurs via inhalation of microconidia from contaminated soil, particularly in areas with bird or bat droppings, where fungal spores can persist for extended periods [1,3]. Infection occurs when microconidia or mycelial fragments are inhaled and deposited in the lungs, causing primary pulmonary histoplasmosis. The host's immune status plays a crucial role in determining the extent of fungal dissemination. In immunocompromised hosts, such as pediatric oncology patients, the infection can disseminate hematogenously or via lymphatics to organs such as the liver, spleen, and bone marrow, resulting in disseminated histoplasmosis [2].

In children with cancer, factors such as prolonged neutropenia, mucosal barrier disruption, corticosteroid use, and chemotherapy increase vulnerability to opportunistic pathogens [9]. Although relatively rare, disseminated histoplasmosis in this population can progress rapidly and may be fatal without timely recognition and treatment [4].

Our patient presented with febrile neutropenia, hepatosplenomegaly, and progressive respiratory compromise, nonspecific symptoms that overlapped with a broad differential diagnosis, including leukemia relapse, tuberculosis, bacterial sepsis, and other fungal infections such as aspergillosis and pneumocystosis [5-7]. As is standard practice, the initial approach focused on bacterial and viral causes of febrile neutropenia. However, persistent fever and clinical deterioration despite broad-spectrum antibacterial and antifungal therapy prompted further diagnostic evaluation.

In immunocompromised children, other invasive fungal infections must be considered. Invasive candidiasis is among the most common, with an incidence of approximately 29 cases per 1,000 children with hematologic malignancies, and a higher risk of dissemination reported with *Candida tropicalis* [10]. Invasive aspergillosis (IA) affects up to 8% of pediatric leukemia patients, particularly those with prolonged neutropenia or corticosteroid exposure, with *Aspergillus fumigatus* being the most frequent species [11]. Mucormycosis, although less frequent, is highly aggressive and associated with high mortality. In a pediatric cohort, 41% of mucormycosis cases occurred in neutropenic children, one-third of whom had hematologic malignancies [12]. In our patient, initial imaging suggested possible pulmonary aspergillosis, leading to empiric voriconazole therapy. However, a negative galactomannan assay and lack of clinical improvement prompted reconsideration, ultimately leading to the identification of *H. capsulatum*.

Diagnosing disseminated histoplasmosis in immunocompromised hosts remains challenging. Blood cultures are often negative or too slow to guide urgent clinical decisions. In pediatric patients, sensitivity ranges from approximately 50% to 75%, depending on fungal burden and immune status [4,8,13]. While histopathology and fungal cultures from tissue biopsies can aid diagnosis, our patient’s ongoing decline led to a critical turning point, reevaluation of the previously collected bone marrow smear. This review revealed intracellular yeast-like forms consistent with *Histoplasma*, which guided appropriate therapy. This case highlights the importance of maintaining a high index of suspicion and the diagnostic value of revisiting earlier specimens when the clinical course is atypical.

Disseminated histoplasmosis can involve the reticuloendothelial system, particularly the liver, spleen, and bone marrow, leading to cytopenias and organomegaly. This presentation can mimic leukemia relapse or even secondary HLH, including histoplasmosis-induced HLH, which, although rare, has been documented in immunosuppressed patients [7]. In our patient, leukemia relapse was initially suspected, but the absence of blasts on bone marrow aspirate and subsequent identification of fungal elements allowed the correct diagnosis to be made.

Epidemiological estimates indicate that histoplasmosis remains a significant public health concern in Central America. A modeling study of HIV-associated histoplasmosis in Latin America reported an incidence of approximately 1.48 cases per 100 persons living with HIV per year, with Central America identified as a high-prevalence region [14]. This translates to substantial morbidity among immunocompromised individuals in Costa Rica and neighboring countries. These data, along with documented outbreaks and pediatric case series from Costa Rica, support the need for heightened clinical awareness in our setting.

Despite Costa Rica being an endemic region for *H. capsulatum*, histoplasmosis remains underrecognized in children. A 1999 outbreak in San José linked to bat cave exposure affected 61 children, with 72% developing acute pulmonary histoplasmosis [15]. More recently, a 10-year retrospective review identified 18 pediatric cases of disseminated histoplasmosis at a national referral hospital, with a mean age of just 12 months [16]. Contributing factors include limited access to specialized diagnostics, nonspecific clinical presentation, and low clinician awareness. In our patient, while no explicit environmental exposure was documented, residence in a rural area likely implied an unrecognized risk. This underscores the need for detailed environmental and geographic history-taking in endemic areas and a low threshold for suspecting histoplasmosis.

Although *Histoplasma* antigen detection in urine or serum is highly sensitive (>90% in disseminated disease) [6], it was not initially ordered due to the early presumptive diagnosis of viral febrile neutropenia and the absence of clear clinical or radiologic suspicion for endemic fungal infection. As the patient worsened, urine antigen testing and PCR from a second bone marrow aspirate were obtained. The antigen result returned within 24 hours, expediting diagnosis and guiding treatment. This sequence illustrates common diagnostic delays and reinforces the importance of considering endemic mycoses early in high-risk patients.

A key learning point is the importance of reexamining diagnostic material when the clinical trajectory deviates from expectations. In this case, reevaluation of the bone marrow smear led to the definitive diagnosis and appropriate treatment. Such flexibility, combined with close collaboration between clinicians and laboratory teams, is essential in managing complex immunocompromised patients.

In the literature, median time from symptom onset to diagnosis in pediatric patients with disseminated histoplasmosis ranges from 14 to 21 days [7]. Longer delays are associated with worse outcomes. Our case followed a similar timeline, but early recognition based on reanalysis of existing specimens may have positively influenced the outcome.

Although β-D-glucan levels were markedly elevated (>500 pg/mL), this test lacks specificity for *Histoplasma* and may also be positive in other fungal infections such as candidiasis and aspergillosis. It should be interpreted in conjunction with specific diagnostic tests and clinical context [17].

Liposomal amphotericin B is the treatment of choice for severe or disseminated histoplasmosis in immunocompromised hosts due to its fungicidal activity and better safety profile compared to amphotericin B deoxycholate [9]. After initial therapy, itraconazole is recommended for at least 12 months, with treatment duration tailored to immune recovery and clinical response. In our patient, amphotericin B led to rapid clinical improvement, with resolution of respiratory distress and hospital discharge after two weeks of IV therapy followed by oral itraconazole. Notably, treatment was complicated by hypokalemia, a known adverse effect requiring careful monitoring.

## Conclusions

Disseminated histoplasmosis, while rare, must be considered in the differential diagnosis for persistent neutropenia in immunocompromised pediatric patients, especially those living in endemic areas. This case highlights the critical need to elevate clinical suspicion when conventional treatments fail and standard diagnostics are inconclusive. Bone marrow involvement may closely mimic leukemia relapse, underscoring the importance of detailed morphologic review and the use of molecular diagnostics such as PCR for definitive diagnosis. While non-invasive fungal biomarkers and imaging can aid in guiding clinical suspicion, they often lack the specificity needed for a definitive diagnosis. Early initiation of targeted antifungal therapy, particularly liposomal amphotericin B followed by itraconazole, remains the cornerstone of management in severe cases. Ultimately, timely diagnosis and multidisciplinary collaboration can dramatically change outcomes, significantly improving outcomes in what can be a life-threatening infection when prompt diagnosis and treatment are achieved. 
